# Pressure ulcer-related pelvic osteomyelitis: evaluation of a two-stage surgical strategy (debridement, negative pressure therapy and flap coverage) with prolonged antimicrobial therapy

**DOI:** 10.1186/s12879-018-3076-y

**Published:** 2018-04-10

**Authors:** Johan Andrianasolo, Tristan Ferry, Fabien Boucher, Joseph Chateau, Hristo Shipkov, Fatiha Daoud, Evelyne Braun, Claire Triffault-Fillit, Thomas Perpoint, Frédéric Laurent, Alain-Ali Mojallal, Christian Chidiac, Florent Valour, on behalf of the Lyon BJI study group, Tristan Ferry, Florent Valour, Thomas Perpoint, André Boibieux, François Biron, Patrick Miailhes, Florence Ader, Agathe Becker, Sandrine Roux, Claire Triffault-Fillit, Fatiha Daoud, Johanna Lippman, Evelyne Braun, Christian Chidiac, Yves Gillet, Laure Hees, Sébastien Lustig, Elvire Servien, Yannick Herry, Romain Gaillard, Antoine Schneider, Michel-Henry Fessy, Anthony Viste, Philippe Chaudier, Romain Desmarchelier, Tanguy Mouton, Cyril Courtin, Lucie Louboutin, Sébastien Martres, Franck Trouillet, Cédric Barrey, Francesco Signorelli, Emmanuel Jouanneau, Timothée Jacquesson, Ali Mojallal, Fabien Boucher, Hristo Shipkov, Mehdi Ismail, Joseph Chateau, Frédéric Aubrun, Isabelle Bobineau, Caroline Macabéo, Frederic Laurent, François Vandenesch, Jean-Philippe Rasigade, Céline Dupieux, Fabien Craighero, Loic Boussel, Jean-Baptiste Pialat, Isabelle Morelec, Marc Janier, Francesco Giammarile, Michel Tod, Marie-Claude Gagnieu, Sylvain Goutelle, Solweig Gerbier-Colomban, Thomas Benet, Eugénie Mabrut

**Affiliations:** 1 Department of infectious diseases, Hospices Civils de Lyon, Lyon, France; 2 CRIOAc Lyon, Regional reference center for the management of complex bone and joint infection, Hospices Civils de Lyon, Lyon, France; 3 Department of general medicine, Claude Bernard Lyon University, Lyon, France; 4 CIRI – Centre International de Recherche en Infectiologie, Inserm, U1111, Université Claude Bernard Lyon 1, CNRS, UMR5308, Ecole Normale Supérieure de Lyon, Univ Lyon, F-69007 Lyon, France; 5 Department of plastic, reconstructive and aesthetic surgery, Hospices Civils de Lyon, Lyon, France; 6 Laboratory of bacteriology, French national reference center for staphylococci, Hospices Civils de Lyon, F-69007 Lyon, France; 7 Service des maladies infectieuses et tropicales, Centre de Référence inter-régional pour la prise en charge des Infections Ostéo-Articulaires complexes (CRIOAc), Hôpital de la Croix-Rousse, 103 Grande-Rue de la Croix-Rousse, 69004 Lyon, France

**Keywords:** Antimicrobial therapy, Bacteriology, Chronic osteomyelitis, Debridement, Flap coverage, Negative pressure therapy, Pressure ulcer

## Abstract

**Background:**

A two-stage surgical strategy (debridement-negative pressure therapy (NPT) and flap coverage) with prolonged antimicrobial therapy is usually proposed in pressure ulcer-related pelvic osteomyelitis but has not been widely evaluated.

**Methods:**

Adult patients with pressure ulcer-related pelvic osteomyelitis treated by a two-stage surgical strategy were included in a retrospective cohort study. Determinants of superinfection (i.e., additional microbiological findings at reconstruction) and treatment failure were assessed using binary logistic regression and Kaplan-Meier curve analysis.

**Results:**

Sixty-four pressure ulcer-related pelvic osteomyelitis in 61 patients (age, 47 (IQR, 36–63)) were included. Osteomyelitis was mostly polymicrobial (73%), with a predominance of *S. aureus* (47%), *Enterobacteriaceae* spp. (44%) and anaerobes (44%). Flap coverage was performed after 7 (IQR, 5–10) weeks of NPT, with 43 (68%) positive bone samples among which 39 (91%) were superinfections, associated with a high ASA score (OR, 5.8; *p* = 0.022). An increased prevalence of coagulase negative staphylococci (*p* = 0.017) and *Candida* spp. (*p* = 0.003) was observed at time of flap coverage. An ESBL *Enterobacteriaceae* spp. was found in 5 (12%) patients, associated with fluoroquinolone consumption (OR, 32.4; *p* = 0.005). Treatment duration was as 20 (IQR, 14–27) weeks, including 11 (IQR, 8–15) after reconstruction. After a follow-up of 54 (IQR, 27–102) weeks, 15 (23%) failures were observed, associated with previous pressure ulcer (OR, 5.7; *p* = 0.025) and *Actinomyces* spp. infection (OR, 9.5; *p* = 0.027).

**Conclusions:**

Pressure ulcer-related pelvic osteomyelitis is a difficult-to-treat clinical condition, generating an important consumption of broad-spectrum antibiotics. The lack of correlation between outcome and the debridement-to-reconstruction interval argue for a short sequence to limit the total duration of treatment.

## Background

Pressure ulcers are frequent and severe clinical conditions corresponding to localized areas of damaged skin and/or underlying tissues over a bony prominence. As resulting of pressure in combination with shear associated with immobility, these lesions mostly occur in para- or tetraplagic patients after spinal cord injury, or in geriatric or intensive care settings [1, 2]. Stage 4 lesions of the revised national pressure ulcer advisory panel (NPUAP) pressure injury staging system are associated with deep-seated infections, including contiguous osteomyelitis that has been reported in 17 to 32% of patients [3–5]. Local care and/or antimicrobial therapy alone are insufficient to manage these complex infections [6, 7]. Consequently, up to 27% of patients with primary diagnosis of pressure ulcer require a multidisciplinary approach with surgical flap reconstruction and prolonged antimicrobial therapy, leading to massive societal costs approaching 125,000 USD per episode [8–10].

In the absence of formal guidelines, medical and surgical practices are highly heterogeneous, as well as outcomes with failure rates ranging from 5 to 65% [8, 11–14]. Even if immediate reconstruction is proposed by some surgical teams [15], one of the most commonly accepted options for the treatment of sacral or ischial pressure ulcer-related chronic osteomyelitis is a two-stage surgical strategy. The first surgical step consists in debridement of devitalized tissue and allows the realization of multiple bone biopsies aiming for microbiological documentation [2, 16, 17], and is followed by negative pressure therapy (NPT) [18]. A reconstructive surgery is performed after control of the soft tissue infection, commonly using a regional myo- or fascio-cutaneous flap [19]. Antimicrobial therapy is started after the initial surgical debridement, adapted to bacteriological documentation, and prolonged for several weeks after flap coverage. In this context, the present study aimed to relate the experience of a French regional reference center for the management of complex bone and joint infection (CRIOAc Lyon) in such poorly evaluated two-stage surgical strategy, focusing on bacteriological findings and risk factor for treatment failure.

## Methods

### Inclusion criteria and data collection

This retrospective observational monocentric study included all adult patients with ulcer pressure-related sacral or ischial chronic osteomyelitis managed by a two-stage surgical strategy (i.e, debridement followed by NPT before myo- or fascio-cutaneous flap reconstruction) associated with prolonged antimicrobial therapy from January 1st 2012 to April 30th 2016. Patient identification was based on the prospective and exhaustive database of our regional reference center for the management of complex bone and joint infection. For each patient, extensive data were extracted from medical records, nursing charts and biological software, and recorded in a standardized anonymous case report form. Collected data included patients and osteomyelitis baseline characteristics, past medical history allowing the calculation of the modified comorbidity Charlson index as previously described [20], the precise surgical and medical therapeutic sequences, results of microbiological analysis at each surgical step, and outcome.

### Definitions

In the absence formal consensus, the diagnosis of osteomyelitis was based on clinical, radiological and microbiological findings [21]. Histological analysis was not routinely performed and consequently not included in the diagnosis criteria. Reported microbiological findings relied only on gold-standard sample management, consisting in prolonged (14 days) cultures of surgical bone biopsies performed after ulcer debridement. Results of superficial and/or soft tissue samples were excluded. To be considered as implicated in bone infection, potentially contaminant bacteria such as coagulase negative staphylococci (CoNS), *Corynebacteria* spp., or *Propionibacterium* spp. had to be yielded on at least two samples, as suggested by the US guidelines for the diagnosis of prosthetic joint infection [22], and taken into account by the treating clinician in the definitive antibiotic regimen. Superinfection referred to additional microbiological findings at time of flap reconstruction in comparison with debridement. Therapeutic failure included: i) the need for additional surgical procedure for septic reason after flap reconstruction; ii) relapse at the same site after discontinuing antimicrobial therapy; and/or iii) infection-related death.

### Statistical analysis

Descriptive statistics were used to estimate the frequencies of the study variables, described as percentages (%) for dichotomous variables and as medians (interquartile range (IQR)) for continuous values. For each variable, the number of missing values was excluded from the denominator in percentage calculation. Non-parametric statistical methods were used to compare groups (Fisher exact test and Mann-Whitney U test), as appropriate. Kaplan-Meier curves allowed the comparison of failure-free survival between groups using the log-rank test. Determinants of superinfections and treatment failure were assessed using binary logistic regression, and expressed as odd ratio (OR) with 95% confidence interval (95%CI). Clinically pertinent variables with a *p*-value < 0.15 in the univariate analysis were included in the final multivariate models. A *p*-value < 0.05 was considered as significant. All analyses were performed using SPSS software version 19.0 (SPSS, Chicago, IL).

## Results

### Included population

Sixty-four pressure ulcer-related ischial (*n* = 43; 67.2%) or sacral (*n* = 20; 31.3%) osteomyelitis occurring in 61 patients (46 males, 71.9%; median age, 47.4 [IQR, 35.6–62.6]) were included. Contexts leading to pressure ulcer were mostly paraplegia (*n* = 41; 64.1%) and tetraplegia (*n* = 12; 18.8%). Patients had few comorbidities, including 9 (14.0%) with diabetes mellitus and 4 (6.3%) with heart failure or chronic liver disease, leading to a median modified Charlson’s comorbidity index of 3 (IQR, 2–5). Twenty-five (39.1%) were active smokers. A previous pressure ulcer at the same site was noted in 24 (37.5%) patients. A consumption of third generation cephalosporin, piperacilline-tazobactam, carbapenam and/or fluoroquinolone was reported in 8 (12.5%), 11 (17.2%), 11 (17.2%) and 8 (12.5%) cases, respectively. All patients’ characteristics are presented in Table 1.

**Table 1:** Comparison of patients with favorable and unfavorable outcome and determinants of treatment failure

	Total population	Superinfection				Outcome			
		Superinfection	*p*-value^*^	OR (95%CI)	*p*-value	Treatment failure	*p*-value^#^	OR (95%CI)	*p*-value
Demographics									
Male gender	46 (71.9%)	26 (66.7%)	0.242	0.400 (0.113–1.415)	0.155	12 (80.0%)	0.525	1.765 (0.434–7.181)	0.428
Age, years	47.4 (35.6–62.6)	46.3 (34.0–62.9)	0.815	0.987 (0.713–1.365) ^a^	0.935	50.3 (40.4–65.5)	0.521	1 .071 (0.737–1.555) ^a^	0.720
Comorbidities									
BMI, kg/m^2^	23.6 (20.6–26.9)	24.1 (20.6–27.0)	0.784	1.033 (0.926–1.152)	0.560	22.9 (21.7–26.7)	0.869	0.996 (0.881–1.125)	0.948
Albumin, g/L	28.0 (24.3–32.1)	29.5 (26.2–32.0)	0.037	1.106 (0.998–1.226)	0.055	26.5 (23.7–30.0)	0.354	0.959 (0.869–1.059)	0.412
Prealbumin, g/L	0.2 (0.1–0.2)	0.2 (0.1–0.2)	0.285	142 (0.023–895,167)	0.267	0.2 (0.1–0.3)	0.716	0.964 (0.000–6272)	0.993
Diabetes mellitus	9 (14.0%)	5 (12.8%)	1.000	1.029 (0.223–4.760)	0.970	2 (13.3%)	1.000	0.923 (0.170–5.003)	0.926
Chronic renal failure	2 (3.1%)	1 (2.6%)	1.000	0.605 (0.036–10.152)	0.727	1 (6.7%)	0.417	3.429 (0.201–58.391)	0.394
Chronic hepatic disease	4 (6.3%)	2 (5.1%)	0.632	0.595 (0.078–4.526)	0.616	3 (20.0%)	0.037	12.000 (1.145–125.816)	0.038
Congestive heart failure	4 (6.3%)	3 (7.7%)	1.000	1.917 (0.188–19.559)	0.583	1 (6.7%)	1.000	1.095 (0.105–11.380)	0.939
Peripheral artery disease	1 (1.6%)	1 (2.6%)	1.000	NC	NC	0 (0.0%)	1.000	NC	
Cerebral artery disease	2 (3.1%)	2 (5.1%)	0.521	NC	NC	0 (0.0%)	1.000	NC	
Immunodepression	4 (6.3%)	2 (5.1%)	0.632	0.595 (0.078–4.526)	0.616	1 (6.7%)	1.000	1.095 (0.105–11.380)	0.939
Solid tumor or hemopathy	4 (6.3%)	3 (7.7%)	1.000	1.917 (0.188–19.559)	0.583	1 (6.7%)	1.000	1.095 (0.105–11.380)	0.939
Gastroduodenal ulcer	4 (6.3%)	2 (5.1%)	1.000	1.243 (0.107–14.497)	0.862	1 (6.7%)	1.000	1.095 (0.105–11.380)	0.939
Active smoking	25 (39.1%)	14 (35.9%)	0.596	0.662 (0.235–1.864)	0.435	6 (40.0%)	1.000	1.053 (0.323–3.433)	0.932
ASA score	2.0 (2.0–3.0)	3.0 (2.0–3.0)	0.027	2.774 (1.103–6.974)	0.030	2.0 (2.0–3.0)	0.866	0.877 (0.340–2.261)	0.785
Modified CCI	3.0 (2.0–5.0)	3.0 (2.0–5.0)	0.448	1.041 (0.857–1.264)	0.687	2.0 (2.0–4.0)	0.299	1.134 (0.927–1.385)	0.221
Causal disability									
Evolution delay, years	15.4 (7.5–26.2)	14.9 (5.8–31.4)	0.954	1.009 (0.969–1.051)	0.671	16.5 (9.1–26.2)	0.978	0.997 (0.953–1.043)	0.901
Context									
Hemiplegic	2 (3.1%)	1 (2.6%)	1.000	0.605 (0.036–10.152)	0.727	1 (6.7%)	0.417	3.429 (0.201–58.391)	0.394
Paraplégic	41 (64.1%)	25 (64.1%)	1.000	0.893 (0.306–2.607)	0.836	8 (53.3%)	0.366	0.554 (0.171–1.798)	0.326
Quadriplegic	12 (18.8%)	8 (20.5%)	0.509	1.806 (0.429–7.608)	0.420	3 (20.0%)	1.000	1.111 (0.259–4.717)	0.887
Geriatrics	3 (4.7%)	2 (5.1%)	1.000	1.243 (0.107–14.497)	0.862	1 (6.7%)	0.558	1.679 (0.141–19.915)	0.682
ICU	1 (1.6%)	1 (2.6%)	1.000	NC	NC	0 (0.0%)	1.000	NC	
History of previous pressure ulcer									
At the same site	24 (37.5%)	17 (43.6%)	0.295	1.877 (0.635–5.549)	0.255	10 (66.7%)	0.013	5.000 (1.448–17.271)	0.011
Previous surgery at the same site	22 (34.4%)	14 (35.9%)	1.000	1.120 (0.384–3.270)	0.836	9 (60.0%)	0.028	4.154 (1.236–13.960)	0.021
Previous flap at the same site	9 (14.1%)	6 (15,4%)	1.000	1.273 (0.287–5.647)	0.751	4 (26.7%)	0.196	3.200 (0.735–13.938)	0.121
Actual pressure ulcer									
Sacrum	20 (31.3%)	13 (33.3%)	0.787	1.214 (0.403–3.661)	0.730	5 (33.3%)	1.000	1.133 (0.330–3.891)	0.842
Ischium	43 (67.2%)	27 (69.2%)	0.585	1.350 (0.463–3.937)	0.583	10 (66.7%)	1.000	0.970 (0.284–3.312)	0.961
Surgical debridement									
Delay since ulcer onset, weeks	37.9 (14.0–109.6)	36.4 (16.2–131.1)	0.630	1.003 (0.997–1.009)	0.350	41.7 (10.6–132.6)	0.868	1.000 (0.996–1.003)	0.858
CRP level at debridement, mg/L	42.2 (20.6–101.8)	43.0 (20.8–125.0)	0.948			52.5 (17.8–141.1)	0.792	1.001 (0.994–1.009)	0.708
Diverting colostomy	18 (28.1%)	14 (35.9%)	0.152	2.800 (0.796–9.843)	0.108	6 (40.0%)	0.326	2.056 (0.606–6.970)	0.247
Flap closure									
Delay since the last debridement, weeks	6.6 (4.9–9.6)	6.1 (4.8–9.9)	0.938	1.040 (0.946–1.144)	0.419	5.6 (5.1–8.0)	0.657	0.952 (0.843–1.076)	0.430
CRP level at time of flap closure, mg/L	25 (14.7–43.6)	25.5 (14.4–46.0)	0.356	1.008 (0.985–1.031)	0.517	22.9 (15.5–28.1)	0.474	0.879 (0.947–1.012)	0.210
Broad spectrum antimicrobial use before flap closure									
3^rd^GC	14 (21.9%)	9 (23.1%)	1.000	1.140 (0.332–3.920)	0.835	5 (33.3%)	0.286	2.222 (0.609–8.108)	0.227
Piperacillin-tazobactam	40 (62.5%)	21 (53.8%)	0.115	0.389 (0.127–1.190)	0.098	9 (60.0%)	1.000	0.871 (0.266–2.849)	0.819
Carbapenem	16 (25.0%)	10 (25.6%)	1.000	1.034 (0.321–3.335)	0.955	6 (40.0%)	0.173	2.600 (0.749–9.029)	0.132
Fluoroquinolone	18 (28.1%)	8 (20.5%)	0.157	0.430 (0.138–1.337)	0.145	5 (33.3%)	0.744	1.385 (0.398–4.818)	0.609
Vancomycin	44 (68.8%)	27 (69.2%)	1.000	0.926 (0.305–2.818)	0.893	10 (66.7%)	1.000	0.882 (0.257–3.029)	0.842
Antimicrobial therapy									
Intravenous antimicrobial therapy									
Total duration, weeks	15.7 (10.7–22.0)	19.4 (13.1–24.8)	0.021	NA	NA	16.9 (8.1–26.4)	0.687	NA	NA
Duration from flap closure, weeks	7.9 (5.0–12.9)	10.1 (6.9–13.5)	0.008	NA	NA	11.2 (4.4–14.7)	0.386	NA	NA
Relay to oral antimicrobial therapy	24 (38.7%)	11 (29.7%)	0.067	NA	NA	7 (50.0%)	0.363	NA	NA
Total duration of antimicrobial therapy from flap closure, weeks	11.1 (7.5–15.1)	12.0 (8.0–15.1)	0.893	NA	NA	13.4 (11.1–23.4)	0.011	NA	NA
Total duration of antimicrobial therapy, weeks	19.8 (13.8–27.4)	21.0 (13.9–25.2)	0.789	NA	NA	28.9 (17.0–32.1)	0.109	NA	NA
Outcome									
Treatment failure	15 (23.4%)	8 (20.5%)	0.545	0.627 (0.194–2.028)	0.435	15 (100.0%)	NA	NA	NA
Delay since flap closure, weeks	12.4 (7.3–28.3%)	15.9 (11.1–27.3)	0.563	NA	NA	12.4 (7.3–28.3)	NA	NA	NA
Requirement of additional surgical procedure	14 (93.3%)	7 (87.5%)	1.000	NC	NC	14 (93.3%)	NA	NA	NA
Relapse after treatment discontinuation	13 (20.3%)	7 (17.9%)	0.535	0.656 (0.191–2.254)	0.503	13 (86.7%)	NA	NA	NA
Death	4 (6.3%)	3 (7.7%)	1.000	1.917 (0.188–19.559)	0.583	2 (13.3%)	0.232	NC	NC
Infection-related death	2 (50.0%)	1 (33.3%)	1.000	NC	NC	2 (100.0%)	NC	NC	NC

*3* ^*rd*^ *GC, Third generation cephalosporin; 95%CI, 95% confidence interval; ASA, American society of anesthesiologists; BMI, Body mass index; CCI, Charlson comorbidity index; CoNS, Coagulase negative staphylococci; CRP, C-reactive protein; ESBL, Extended spectrum betalactamase: ICU, Intensive care unit; MRCoNS, Methicillin-resistant coagulase negative staphylococci; MRSA, Methicillin-resistant Staphylococcus aureus; NA, Not applicable; NC, Not calculable; OR, Odd ratio*

* *In comparison to patients with no superinfection (Fisher exact test or Mann Whitney U-test, as appropriate)*

^#^ *In comparison to patients with no treatment failure (Fisher exact test or Mann Whitney U-test, as appropriate)*

^a^ *Calculated for 10 additional years*

### Debridement

Lesions evolved from a median of 37.9 (IQR, 14.0–109.6) weeks before debridement. Fifty-seven (89.1%) cases required only one surgical debridement before reconstruction, while 7 (10.9%) had at least two debridements.

**Fig. 1:**
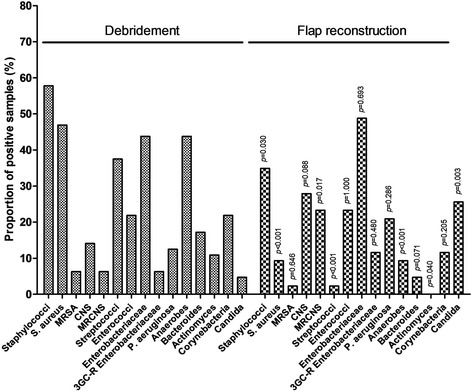
Comparison of microbiological findings among bone biopsies performed at debridement and flap reconstruction

The majority of infections were polymicrobial (*n* = 47; 73.4%). Staphylococci were the most prevalent pathogens, isolated in 37 (57.8%) cases, and including: i) *S. aureus* (*n* = 30; 46.9%), among which 4 (13.3%) were methicillin-resistant (MRSA); and ii) CoNS (*n* = 9; 14.1%) among which 4 (44.4%) were methicillin-resistant (MRCoNS). Other bacterial species included 28 (43.8%) *Enterobacteriaceae* spp. among which 4 (14.8%) secreted extended spectrum betalactamase (ESBL), anaerobes (*n* = 28, 43.8%, including 7 *Actinomyces* spp. [10.9%]), and streptococci (*n* = 24; 37.5%). All microbiological results are detailed in Fig. 1.

Empiric antimicrobial combinations mostly comprised vancomycin (*n* = 44; 69.8%), associated with piperacillin-tazobactam (*n* = 36; 57.1%) or carbapenem (*n* = 11; 22.2). Retrospectively, 82.5% were effective against the pathogens isolated from debridement bone biopsies. Antibiotic therapy was adapted to microbiological results before the flap closure in 49 (77.8%) patients (Table 2).

**Table 2:** Large spectrum antibiotics used during the whole therapeutic sequence

	Empirical antimicrobial therapy after debridement	Antimicrobial therapy adaptation according to debridement samples microbiological results	Re-broadening spectrum of antimicrobial therapy after flap closure	Antimicrobial therapy adaptation according to flap samples microbiological results
3^rd^GC	5 (8.2%)	10 (15.9%)	10 (15.6%)	18 (28.1%)
Piperacillin-tazobactam	36 (57.1%)	22 (39.4%)	20 (31.3%)	10 (15.6%)
Carbapenem	14 (22.2%)	8 (12.7%)	11 (17.2%)	9 (14.1%)
Vancomycin	44 (69.8%)	26 (41.3%)	25 (39.1%)	23 (35.9%)
Fluoroquinolone	7 (11.1%)	15 (24.2%)	23 (35.9%)	26 (40.6%)

### Flap reconstruction

The flap reconstruction was performed 6.6 (IQR, 4.9–9.6) weeks after debridement. Spectrum of antibiotic therapy was empirically re-expanded after the reconstruction step in 15 (23.1%) patients (Table 2).

Bacterial cultures of bone biopsies were positive in 43 (68.3%) cases. Fourteen (21.9%) patients had at least one bacteria already present in initial debridement bone samples. These persisting infections were due to *Enterobacteriaceae* spp. (n = 4), CoNS (n = 3), *Corynebacteria* spp. (n = 3), MSSA (n = 2), *P. aeruginosa* (n = 2), *E. faecalis* (n = 1), *Finegoldia* spp. (n = 1) and *C. tropicalis* (n = 1). However, patients with positive bone samples at time of flap reconstruction mostly had a superinfection (*n* = 39; 90.7%). Characteristics of patients with superinfection and univariate analysis for its determinants are provided in Table 1. In multivariate analysis, the only independent risk factor of superinfection was the ASA score (OR, 5.758; 95%CI, 1.284–25.833; *p* = 0.022). Post-debridement appropriate empiric antibiotic therapy was protective (OR, 0.069; 95%CI, 0.006–0.787; *p* = 0.031).

Compared to the initial bacteriological findings (Fig. 1), staphylococci were globally less represented (23.4%; *p* = 0.030), with a decrease in *S. aureus* prevalence (9.3%; *p* < 10^− 3^). Contrariwise, CoNS were increasingly found in 12 (27.9%) cases (*p* = 0.017) among which 83.3% were methicillin-resistant, without identified risk factor for MRCoSN superfinfection. A significant reduction in the proportion of streptococci (2.3%; *p* < 10^− 3^) and anaerobes (9.3% without any *Actinomyces* spp.; *p* < 10^− 3^) was observed. An ESBL-producing *Enterobacteriaceae* spp. was found in 11.6% of cases compared to 6.3% at the time of trimming (*p* = 0.480), such a superinfection being statistically associated with the use of fluoroquinolones in the previous 6 months (OR, 32.4; 95%CI, 2.820–372.319; *p* = 0.005). *Pseudomonas aeruginosa* superinfection (*n* = 7; 17.9%) was associated with a high modified Charlson’s comorbidity index (OR, 1.269; 95%CI, 0.997–1.614; *p* = 0.053) and multiple debridements (OR, 7.067; 95%CI, 0.946–52.766; *p* = 0.057). An increase in the prevalence of *Candida albicans* was finally observed (25.6% vs. 4.7%; *p* = 0.003), without identified predictive factor with respect of male sex (OR, 0.229; 95%CI, 0.053–0.987; *p* = 0.048). In particular, the use of broad spectrum betalacam antibiotic, including carbapenem (OR, 0.816; 95%CI, 0.151–4.403; *p* = 0.813), was not associated with fungal superinfection.

### Antimicrobial therapy

All patients were initially treated intravenously; an oral switch could be possible for 24 (38.7%) of them, only. The total duration of antimicrobial therapy was 19.8 (IQR, 13.8–27.4) weeks, including 11.1 (IQR, 7.5–15.1) weeks after flap reconstruction. In patients with fungal infection and/or superinfection, antifungal drugs were prolonged for 25.9 (IQR, 15.3–26.9) weeks after flap reconstruction.

### Outcome

**Fig. 2:**
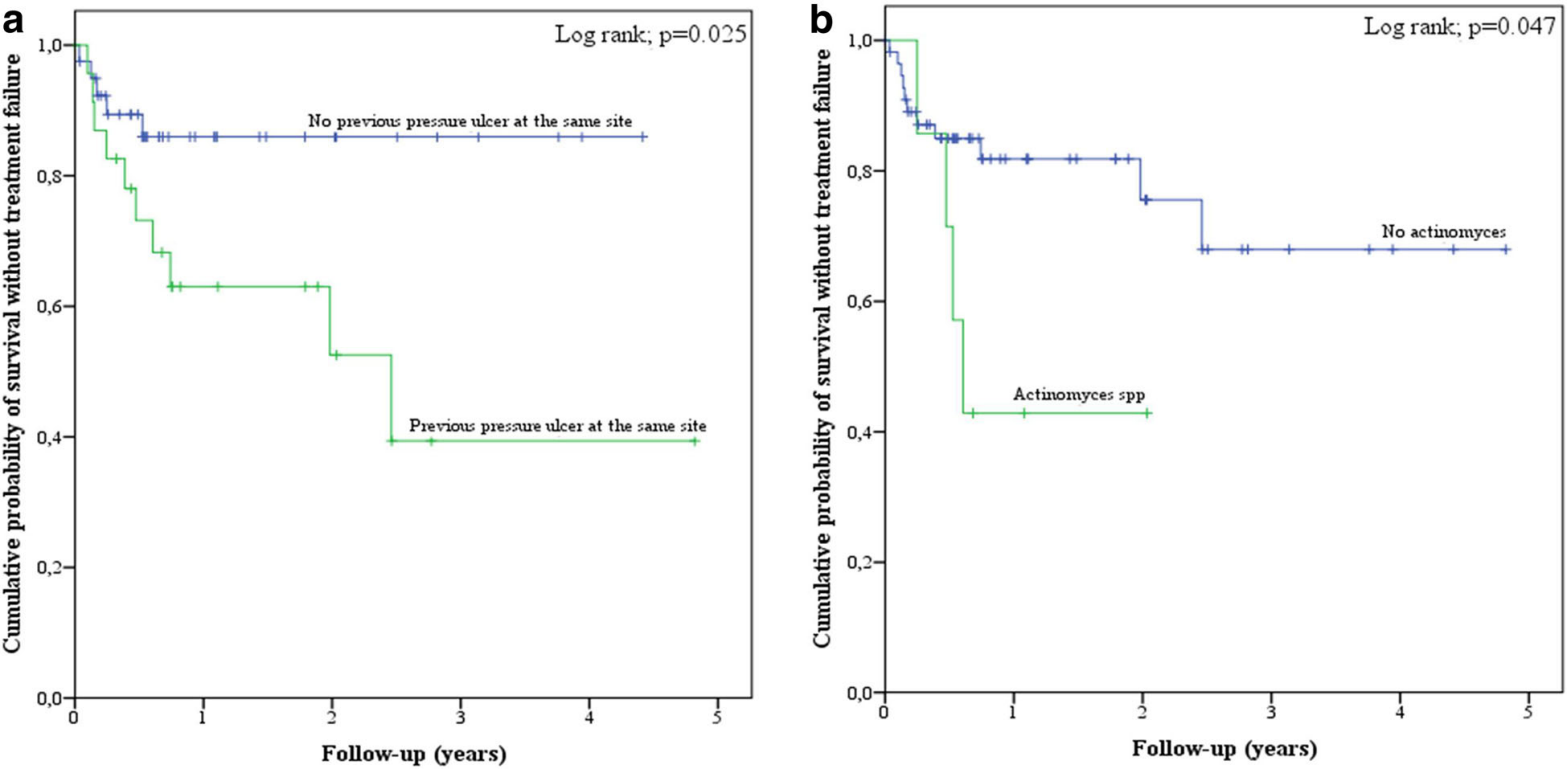
Kaplan Meier curves for cumulative probability of treatment failure-free survival according to the two main risk factors highlighted in multivariate analysis, i.e., an history of previous ulcer at the same site (**a**) and *Actinomyces* spp. infection (**b**)

Patients were followed-up for 59.1 (IQR, 37.1–121.3) weeks after debridement, 54 (IQR, 26.6–101.7) weeks after flap reconstruction, and 38.6 (IQR, 13.6–91.1) weeks after antimicrobial interruption. Fifteen (23.4%) treatment failures were diagnosed in a median delay of 12.4 (IQR, 7.3–28.3) weeks after flap coverage, necessitating an additional surgical procedure in 14 (93.3%) cases. Four patients died, including 2 deaths related to pressure ulcer-related infection. The diagnosis of treatment failure led to an increased in total antimicrobial therapy length from 9.7 (IQR, 6.8–13.6) weeks to 13.4 (IQR, 11.1–23.4) weeks (*p* = 0.011). The whole comparison between patients with favorable outcome and treatment failure, and risk factors for poor outcome (univariate analysis) are presented in Table 1. In multivariate analysis, independent determinants of treatment failure were the existence of a previous pressure ulcer located at the same site (OR, 5.701; 95%CI, 1.244–26.127; *p =* 0,025) and *Actinomyces* spp.-positive cultures at time of debridement (OR, 9.522; 95%CI, 1.290–70.296; *p =* 0,027). Results of failure-free survival curves analysis are presented in Fig. 2. Colostomy (*n* = 18; 28.1%), the delay between debridement and flap reconstruction, and admission in a rehabilitation center after the debridement (*n* = 16; 25.0%) and/or the flap coverage (*n* = 39; 60.9%) did not influence outcome.

## Discussion

Chronic osteomyelitis complicating end-stage pressure ulcers represent severe clinical conditions with poorly investigated management. This retrospective series provides interesting insights regarding the management of these complex bone infections, including among microbiological diagnosis and outcome of a two-stage surgical strategy in a referral center.

Diagnosis of pressure ulcer-related osteomyelitis is puzzling. Clinical assessment is often inaccurate [5], and no imaging techniques allow an acceptable discernment between osteomyelitis and pressure-related bone change, including magnetic resonance imaging [21, 23]. Histological analysis of bone biopsies does not appear to be more helpful [21]. For the microbiologist, the challenge is to distinguish between colonizing and invasive bacteria, as both originate from the commensal cutaneous and digestive flora. In the absence of validated discriminant criteria, we used a practice-based approach, considering only: i) the results of the bacteriological analysis of surgical bone biopsies sampled after the debridement step, with the exclusion of superficial and/or soft tissues samples [24]; and ii) virulent pathogens (i.e. *S. aureus*, *Pseudomonas* spp. …), and potentially contaminants if yielded on at least two samples as suggested by the US guidelines for the diagnosis of prosthetic joint infection [22], and taken into account by the treating clinician in the definitive antimicrobial regimen. The bacterial distribution observed in our study was consistent with other similar investigations, with a predominance of MSSA, streptococci, *Enterobacteriaceae* spp. and anaerobes [6, 21, 25]. While the diffusion of MRSA is actually controlled in Europe, ESBL-producing *Enterobacteriaceae* spp. are frequently implicated in pressure injury colonization and deep tissue infection, reaching 11.6% at the flap coverage step in our study. They have been associated with wound management in long-term care facilities, particularly in case of fluoroquinolone use [26], as well as highlighted by our results.

The management of chronic pelvic osteomyelitis requires a multidisciplinary approach, with comprehensive assessment of the patient’s general medical condition, proper positioning with four to six weeks of pressure off-loading on adapted support surfaces, optimized nutrition and psychosocial support [15, 27, 28]. A combined medical-surgical approach is mandatory, allowing a better outcome in case of osteomyelitis [6]. In this setting, multidisciplinary staff meetings in referral centers have not been evaluated in the specific field of pelvic osteomyelitis, but have demonstrated their advantages in orthopedic infections [29, 30]. In our center, they have made possible the crucial coordination of all the actors of the patients’ care, including the infectious disease specialists, orthopedic and plastic surgeons, microbiologists, radiologists, nuclear medicine specialists and rehabilitators. They allow an interdisciplinary decision for each complex patient case, with planning of the entire therapeutic sequence at the beginning of the patient management.

Concerning surgical strategy, some authors support a one-stage approach with immediate flap reconstruction even in case of local contamination [15]. However, among the 101 patients included in this study, bone biopsies were performed in 70% of cases and half were positive, only, so that the majority of patients were not suspected to have chronic osteomyelitis, even when it is a well-known risk factor for flap coverage failure [31, 32]. Additionally, a single-stage surgery does not allow the adaptation of the empiric antimicrobial therapy to microbiological results before flap closure; although an inappropriate initial treatment is associated with an over-risk of failure. Consequently, we believe that a first step of debridement is essential for the reduction of bacterial inoculum by necrotic tissues excision and adequate sequestrectomy, and for the realization of gold-standard bacterial samples [28, 33, 34].

Regarding antimicrobial therapy, the polymicrobial nature of pelvic osteomyelitis lead to a greater need of broad spectrum antimicrobials than in other bone infections [7]. An empiric combination of a broad-spectrum betalactam (i.e. piperacillin-tazobactam, or cefepim with metronidazole) and vancomycin can be proposed, thus targeting the most frequently involved microorganisms. An empiric prescription of a carbapenem might be only proposed to patient with high-risk of ESBL-producing *Enterobacteriaceae* spp., including those who had taken fluoroquinolones in the previous 6 months, as suggested by the determinants for ESBL-producing *Enterobacteriaceae* spp. superfinfection highlighted by our results.

As the delay between debridement and flap reconstruction does not appear to influence outcome, a short sequence can be proposed in order to reduce the length of antimicrobial therapy. An interval of 2 to 3 weeks between the two surgical steps could be reasonably proposed, allowing: i) the assessment of the evolution of the soft tissue condition; and ii) the adaptation of the antimicrobial spectrum to the definitive bacteriological culture results (requiring 2 weeks), if necessary. With regard of the high frequency of superinfections, broad spectrum antimicrobial therapy should be prolonged until the definitive culture results of the bacteriological sample performed during flap coverage which necessitate two additional weeks. A more targeted treatment – possibly relying on oral molecules with acceptable bone diffusion if available – should then be proposed for 4 additional weeks, leading to a total duration not exceeding 6 weeks after flap coverage as proposed in most similar studies [6, 35, 36], and more generally in adult chronic osteomyelitis [37, 38], in the absence of fungal or *Actinomyces* spp. infection that require at least 6 months of treatment [39, 40].

Pressure ulcer-related osteomyelitis outcome is poor, with an overall failure rate approaching 25% in our series. Some studies reported lower failure rates, but are associated with important bias: i) most of them used less stringent criteria for defining failure; ii) they not exclusively included patients with osteomyelitis; and iii) most had a shorter length of follow-up. For example, in a North-American study based on a national surgery database, flap coverage of pressure ulcer was associated with a recurrence rate of 1.9% with 4.7% of iterative surgical procedure but in a delay of 30 days, only [8]. However, we demonstrated that the diagnosis of failure usually occur later, in a median of 3 months after flap coverage, mostly necessitating reoperation. In a comparable series, Brunel et al. noted initial and final healing rates of 42% and 37%, only, with a relapse rate of 18% [21]. Other studies with extensive length follow-up showed similar results [11, 15, 32, 41]. Additionally, our institution is a labeled referral center for the management of complex bone and joint infection, leading to the recruitment of the most complex – and consequently the most at-risk of failure – situations, which can explain such a high treatment failure rate.

Risk factors for treatment failure are poorly known. Multiple pressure ulcers occur in more than one-third of patients, and constitute in our series a risk factor for treatment failure, as already suggested by two previous studies [11, 14]. To date, no bacteriological factor had been related to treatment failure. Interestingly, we highlighted an increased risk of failure in *Actinomyces* spp. osteomyelitis, which is a difficult-to-treat anaerobic bacteria that should not be considered as a contaminant and contrariwise requires prolonged (≥ 6 months) antimicrobial therapy [40]. Other previously described risk factors for treatment failure include ischial location, poor diabetes control, impaired nutrition status, active smoking, corticosteroid use and cardiovascular disease [11, 14, 32, 42–45]. The benefit of colostomy is still debated. No impact has been highlighted in our series regarding the risk of superinfection or treatment failure. Additionally, this procedure is at-risk of complications in frail patients and of questionable efficacy [46]. On the other hand, it provides a dry and clean environment that should theoretically limit the risk of fecal contamination of the debrided ulcer and promote flap healing [47].

This study is subject to some limitations, inherent to its retrospective and unicentric nature. In particular, the small sample size and the lack of controls restricts the interpretation of outcome data, event if suggesting interesting insights toward the management of pelvic osteomyelitis. However, larger and controlled evaluation are now mandatory to refine the comprehension and management of these complex bone infections.

## Conclusions

Pressure ulcer-related pelvic osteomyelitis is associated with a high risk of treatment failure despite a complex surgical management and an important consumption of broad-spectrum antimicrobials, thus requiring a collaborative medical-surgical management driven by a trained multidisciplinary team in a reference center. A two-step surgical strategy (debridement – NPT – flap reconstruction) can be proposed, although a short interval (2–3 weeks) between the two procedures might be sufficient, allowing improvement of soft tissue conditions and prospective adaptation of empirical antimicrobial therapy, without excessively lengthening the total duration of treatment.

## Abbreviations

95%CI: 95% Confidence interval
ASA: American society of anesthesiologists
CoNS: Coagulase negative staphylococci
ESBL: Extended spectrum betalactamase
IQR: Interquartile range
MRCoNS: Methicillin-resistant coagulase negative staphylococci
MRSA: Methicillin-resistant *Staphylococcus aureus*
MSCoNS: Methicillin-susceptible coagulase negative staphylococci
MSSA: Methicillin-susceptible *Staphylococcus aureus*
NPT: Negative pressure therapy
NPUAP: National pressure ulcer advisory panel
OR: Odd ratio.
